# Uniaxial Cyclic Stretch Promotes Osteogenic Differentiation and Synthesis of BMP2 in the C3H10T1/2 Cells with BMP2 Gene Variant of rs2273073 (T/G)

**DOI:** 10.1371/journal.pone.0106598

**Published:** 2014-09-05

**Authors:** Jia-mou Li, Yao Zhang, Yuan Ren, Bao-ge Liu, Xin Lin, Jiang Yang, Hu-cheng Zhao, Ya-jie Wang, Lei Song

**Affiliations:** 1 Department of Orthopedics, Beijing Tian Tan Hospital, Capital Medical University, Dongcheng District, Beijing, China; 2 CoreLaboratory for Clinical Medical Research, Beijing Tian Tan Hospital, Capital Medical University, Dongcheng District, Beijing, China; 3 Department of Engineering Mechanics, Tsinghua University, Haidian District, Beijing, China; University of Sheffield, United Kingdom

## Abstract

Ossification of the posterior longitudinal ligament of the cervical spine (OPLL) is characterized by the replacement of ligament tissues with ectopic bone formation, and this result is strongly affected by genetic and local factors. Two single nucleotide polymorphisms (SNPs) of rs2273073 (T/G) and rs235768 (A/T) of bone morphogenetic protein 2 (BMP2) gene which are associated with OPLL have been reported in our previous report. In this study, we confirmed the connection in 18 case samples analysis of BMP2 gene in OPLL patients; additionally, it was also shown from the OPLL patients with ligament tissues that enchondral ossification and expression of BMP2 were significantly higher compared with the non-OPLL patients by histological examination, immunohistochemistry and Western blotting analysis. To investigate the underlying mechanism, we studied the effect of SNPs in cell model. The C3H10T1/2 cells with different BMP2 gene variants were constructed and then subjected to uniaxial cyclic stretch (0.5 Hz, 10% stretch). In the presence of mechanical stress, the expression of BMP2 protein in C3H10T1/2 cells transfected by BMP2 (rs2273073 (T/G)) and BMP2 (rs2273073 (T/G), rs235768 (A/T)) were significantly higher than the corresponding static groups (*P*<0.05). In conclusion, these results suggested that BMP2 gene variant of rs2273073 (T/G) could not only increase cell susceptibility to bone transformation similar to pre-OPLL change, but also increase the sensibility to mechanical stress which might play an important role during the progression of OPLL.

## Introduction

Ossification of the posterior longitudinal ligament (OPLL) is a kind of abnormal calcification of the posterior longitudinal ligament and the most affected location is at the cervical spine region which may compress the spinal cord and roots, at the same time, lead to various degrees of neurological symptoms from discomfort to severe myelopathy. It is a common disease in China and throughout Asia [1], [2]. Although the mechanism of OPLL remains unclear, genetic and local factors have been proposed and partly confirmed.

Recent molecular genetic studies identified several candidate genes contributed to this disease. These genes include Core binding factor alpha 1 (Cbfa1) [3], endothelin-1 (ET-1) [4], parathyroid hormone (PTH) [5], prostaglandin I2 (PGI2) [6], connexin43 (Cx43) [7], retinoic X receptor [8], collagen 6A1 (COL6A1) and COL11A2 [8]–[11], transforming growth factor-bate 1 (TGF-β1) [12], [13], TGF3 [14] and nucleotide pyrophosphatase (NPPS) [15]. However, none of these genes has been confirmed as being pathogenetically relevant for OPLL patients. BMP2 is our leading candidate and already used in clinic as osteoinductive molecules. Numerous studies have shown that BMP2 stimulates osteoblast differentiation in human stem cells [16], cultured rat calvarial osteoblasts [17], ST2 bone marrow stromal cell lines [18], SaOS-2 cells [19] and also OPLL cells [20], leading to the notion that it is involved in the etiology of OPLL development. Accumulating evidences indicated that BMP2 played an important role in the development of OPLL. In our previous study, we successfully identified two missense SNPs of rs2273073 (T/G) and rs235768 (A/T) in the BMP2 gene were significantly associated with OPLL [21], [22] from DNA sequencing analysis and association studies.

It is apparent from the clinical evidences that mechanical stress is one of the local factors that play an important role in OPLL progression which is highly correlated with abnormal strain distribution and mechanical factors [9], [23]–[25]. OPLL is highly progressed after posterior decompressive surgery both in the longitudinal direction and in the thickness, regardless of the ossification type, which suggests that local factors of the cervical spine are significantly important in the progression [26], [27]. The progression of OPLL is highly correlated with abnormal strain distribution in the intervertebral discs and frequently observed when strain in the tensile direction is distributed over the disc [28]. The ossification tends to progress in patients with high mobility. In these patients, the range of motion of the cervical spine was severely limited, indicating that dynamic factors were important in the development of OPLL. Furthermore, in patients that underwent anterior interbody fusion, the development of ossification evidently slowed and even stopped [29]. It is necessary to take into consideration in the treating of this disease that the natural course of the disease and to identify the involvement of static factors, such as compression caused by ossification as well as the dynamic factors [30].

Furthermore, in vitro studies, cyclic stretch induces expression levels of BMP2 and its receptor in OPLL cells [25], [31]. However, the details about the BMP2 gene variants involved in the ossification process stimulated by mechanical stress are still unknown. As we all know, single nucleotide polymorphisms (SNPs) are the most abundant resources of genetic variation among individuals of species, exploring the genetic causes of phenotypic variation in humans still remains a great challenge for human genetics. The C3H10T1/2 cell, a multipotent cell line, has been utilized as a surrogate to study molecular mechanisms of stem cell commitment and differentiation to osteoblasts, adipocytes and chondrocytes [32]. Furthermore, the C3H10T1/2 cell was chosen as the alternative to study OPLL in previous study [21]. In this study, we constructed the C3H10T1/2 cells model with different BMP2 gene variants of rs2273073 (T/G) and/or rs235768 (A/T) and used the stretch apparatus which could apply mechanical stress to assess whether there are differences of sensibility to mechanical stress between the cells model with different BMP2 gene variants.

## Materials and Methods

### Disease criteria and spinal ligament samples

Between January 2013 and September 2013, 18 patients presenting with OPLL and 18 patients with non-OPLL were selected for this study. The diagnosis of OPLL was based on radiological findings including radiographs, computed tomogram (CT), and magnetic resonance imaging (MRI) of the cervical spine according to the criteria reported by Tsuyama [33]. The spinal ligaments were collected aseptically from the 18 OPLL patients during anterior decompressive surgery for decompressing the spinal cord for myelopathy. Meantime, spinal ligaments were taken from 18 non-OPLL patients during surgery for cervical disc herniation and cervical spondylotic myelopathy as control. The study protocol was approved by the ethical committee of Beijing Tian Tan Hospital, Capital Medical University and written informed consent was obtained from all participants before the study.

### Amplification and genotyping of SNPs within the BMP2 gene

Genomic DNA was isolated using Wizard Genomic DNA Purification Kits (Promega, USA) from each individual included in the study. The entire BMP2 coding sequence was amplified in overlapping fragments of 300–600 base pairs by polymerase chain reaction (PCR). In terms of the BMP2 gene, several SNPs were already reported [21], [22]. We analyzed rs2273073 (T/G) and rs235768 (A/T) among 11 SNPs within BMP2 gene that have been previously described. Primers were designed in close proximity to the selected SNPs which are summarized in Table 1 and Table 2; the sequences of the BMP2 gene were obtained from http://www.ncbi.nim.nih.gov. The PCR products including the SNPs were genotyped by direct sequencing using BigDye Terminator cycle sequencing on an ABI 3730XL POP7 DNA sequencing analysis 5.2 (Applied Biosystems, USA).

**Table 1:** Primers used to amplify the human BMP2 gene.

Primers	Sense	Antisense	Product size(bp)	Annealing temperature(°C)
Primer 1	GCGTTGGATG GGAGCGATAA	GGAAGCTGCGC ACAGTGTTG	566	58
Primer 2	CTCACGTCG GTCCTGTCC	CCCTGCTCCATG CCTCAC	393	61
Primer 3	GTCCTGTCC TTATCACCTC AGCAGAGC	TTCCATCATGG CCAAAAGTTAC TAGCA	393	60

**Table 2:** The Primers Correlated with SNPs of BMP2 gene.

Gene	SNP ID	Primer
BMP2	rs6085676	Primer 1
	rs1049007	Primer 1
	rs2273073	Primer 1
	rs2273074	Primer 2
	rs36105541	Primer 2
	rs79417223	Primer 2
	rs235768	Primer 3
	rs267606060	Primer 3
	rs34183594	Primer 3
	rs111675841	Primer 3
	rs140884062	Primer 3

### Masson Trichrome staining

The surgical specimens of ligament tissues were fixed by immersion in 4% paraformaldehyde for 24 h and decalcified with RapidCal·Lmmuno (Zhongshan, Beijing, China) for 3–7 days at 4°C. The ligaments tissues were then dehydrated through a graded ethanol series and embedded in paraffin. Sectiotns of 4 um were prepared for Masson Trichrome staining using a microtome. After the paraffin sections were deparafinized and rehydrated, they were fixed in Bouin's solution for 2 hour at 37°C, washed in running tap water to remove the yellow color. Sections were stained in Mayer's Haematoxylin for 3 minutes, washed in running tap water, differentiated in acid alcohol, and washed thoroughly in running tap water. Then, the sections were stained with ponceau fuchsin for 10 minutes, washed in distilled water. After that, the sections were treated with 2% phosphomolybdic acid for 10 minutes, then transferred directly (without rinse) to aniline blue and stained for 5 minutes, treated with acetic acid for 2 minutes, and then dehydrated through a graded series of alcohols, cleared in xylene, and mounted in resinous mounting medium. Masson stainings were used to observe the change of ligaments structure using optical microscopy.

### Immunohistochemistry

The surgical specimens of ligament tissues were disposed as above. Immunohistochemical staining was performed by an EnVision two-step plus method using a two-step plus kits (Zhongshan, Beijing, China). After the paraffin sections were deparafinized and rehydrated, they were incubated in deionized water containing 0.3% hydrogen peroxide for 10 min at room temperature to block endogenous peroxidas. After washed with phosphate buffer salt (PBS), they were incubated in Tris-buffered saline with 0.1% Tween 20 (TBST) containing 10% normal goat serum for 20 min at room temperature to block nonspecific protein binding. The sections were irradiated three times with ethylene diamine tetraacetic acid (EDTA)-sodium citrate, using a microwave oven, for antigen retrieval. Then the sections were reacted with an anti-BMP2 antibody (Abcom, HK), diluted 1∶300 with TBST, at 4°C overnight. They were then incubated with reagent I Polymer Helper for 20 min at room temperature, and finally with reagent II polyperxidase-anti-rabbit IgG for 30 min at room temperature. Color was developed using 3,3-diaminobenzidine tetrahydrochloride containing hydrogen peroxidase. The nuclei were lightly counterstained with hematoxylin. For the control, the same fashion was carried out.

### Western blotting analysis for the surgical specimens of ligament tissues

Ligaments harvested aseptically from patients during surgery were rinsed with PBS, after surrounding tissues were carefully removed under a dissecting microscope. In all cases the ligaments were extirpated carefully from ossified sites to avoid possible contamination of nonosteogenic cells. Then the liquid nitrogen was added to harden the specimens so that they could be milled sufficiently. Cell lysis buffer (50 mM Tris-HCl (pH 7.4), 150 mM NaCl, 20 mM EDTA, 1% Triton X-100, 1% sodium deoxycholate, and 0.1% SDS, supplemented protease inhibitor) was added and cell lysate was collected. The supernatant was obtained by a centrifugation for 5 min at 120,000 g and quantified by using bicinchoninic acid (BCA) relative to bovine serum albumin (BSA) protein standards according to the manufacturer (Pierce, USA). Equal amounts of total proteins were treated with 1×sample buffer, loaded onto an SDS-polyacrylamide gel on 10% acrylamide gels and electrophoresed. Proteins were electrotransferred to PVDF membranes (Millipore, USA) at 100 V for 2 h. They were blocked using 5% milk in TBST and then hybridized for 1 h with anti-BMP2 antibody (Abcom, HK), diluted 1∶1000 with TBST, at 4°C overnight. This was followed by 1 h with goat anti-rabbit IgG (Cell Signaling, USA), diluted 1∶2000 with TBST. Antibody binding was detected by exposure to enhanced chemiluminescence using Immobilon (Millipore, USA) and then visualized on the FluorChem FC2 (Cell Biosciences, USA). For immunodetection of β-tubulin protein, immunoblotting was performed as described above by using anti-β-tubulin monoclonal antibody (Transgen, Beijing, China). Blots were scanned using Kodak Image Station (Kodak, USA) and the optical densities of the bands relative to β-tubulin with each lane were obtained.

### Cell culture

Embryonic mesenchymal stem cells, C3H10T1/2 cells (obtained from American Type Culture Collection, ATCC) were grown in Minimum Essential medium (MEM, Invitrogen) containing 10% fetal bovine serum (FBS, Invitrogen), 50 U/ml penicillin, and 50 µg/ml streptomycin with 5% CO^2^, at 37°C.

### Cell transfection

For stable transfection, the 10^th^ passage of C3H10T1/2 cells were transfected with pcDNA3.1-empty plasmid, pcDNA3.1-BMP2 (wild-type, WT), pcDNA3.1-BMP2 (rs2273073 (T/G)), pcDNA3.1-BMP2 (rs235768 (A/T)), and pcDNA3.1-BMP2 (rs2273073 (T/G), rs235768 (A/T)) (constructed by Invitrogen corporation, USA) and at last they were identified as wild-type BMP2, mutant-type BMP2 and empty vector. The day before transfection, the C3H10T1/2 cells were passaged using standard cell culture techniques and 1.5×10^4^ cells/cm^2^ were plated in 2 ml of media in wells of a 6 well plate so that cells would be approximately 60–80% confluent on the day of transfection. Four cell culture wells per condition were prepared, treated, and analyzed. Transfections were performed using Lipofectamine 2000 (Invitrogen, USA) as per manufacturer's instructions. Four to six hours after transfection, the medium was replaced with fresh growth medium. The following day, cells were trypsinized and replated into a larger sized tissue culture format. Transfected cells were evaluated for GFP expression with fluorescence inversion microscope system (Leica, GER) and then they were screened with G418 at the density of 800 mg/ml for 14 days and only GFP-positive cells were used for study. Control cells were mock transfected with empty vector and selected in the same fashion.

### Stretch apparatus

**Figure 1:**
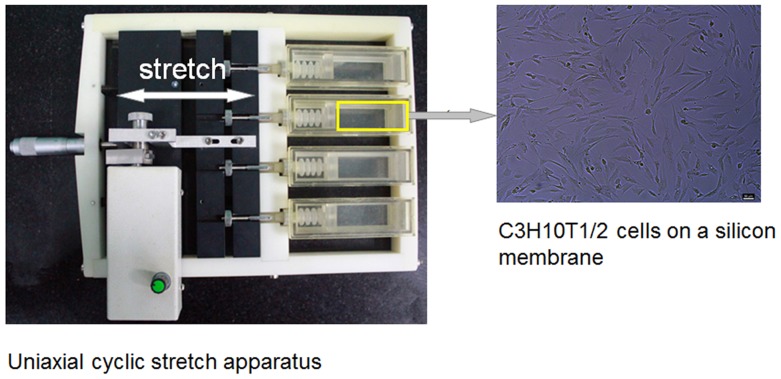
Application of mechanical stress by uniaxial cyclic stretch. The transfected C3H10T1/2 cells were cultured on a deformable silicon membrane coated with gelatin. After reaching confluence, the cells were subjected to uniaxial cyclic stretch. After cyclic stretch, gene expression analyses were performed. In the same conditions, the cells inoculated in the stretch apparatus and not subjected to mechanical stress were as control.

The GFP-positive C3H10T1/2 cells were inoculated on a 2.0×6.0 cm^2^ elastic silicone membrane (Dow Corning, USA) coated with 0.1% gelatin (Zhongshan, Beijing, China) at a density of 10,000 cells/cm^2^. After cultures reached confluence, the cells were incubated in MEM supplemented with 1% FBS for 24 h for synchronization and then subjected to a home-made stretch apparatus (provided by Tsinghua University, Beijing, China) (Fig. 1) in 110% peak to peak, at 0.5 Hz in a humidified atmosphere of 95% air and 5% CO2 at 37°C for 24 h. In the same conditions, the cells inoculated in the stretch apparatus and not applied to mechanical stress were as control groups.

### Morphology analysis of the transfected C3H10T1/2 cells

We observed the morphology changes of the transfected C3H10T1/2 cells before the application of mechanical stress and again at 24 h. The morphology changes were observed through white light vision and the same view was evaluated for GFP expression with fluorescence inversion microscope (Leica, GER).

### Western blot analysis for the transfected C3H10T1/2 cells

We evaluated the expression of BMP2 before the application of mechanical stress and again at 24 h. The C3H10T1/2 cells were washed twice with PBS and the cell lysis buffer was added and then collected. The supernatant was obtained by a centrifugation for 5 min at 120,000 g and quantified by using BCA relative to BSA protein standards according to the manufacturer (Pierce, Rockford, IL, USA). The specific operation steps were as above and replicated for 3 times.

### Statistical analysis

All data were expressed as the mean ± SD. ANOVA with the Dunnett type test for multiple comparisons against a control and Student's test was used. The genotypic and allelic distributions were evaluated using Chi-square test. *P*<0.05 was considered statistically significant. All statistical analyses were performed with the SPSS 17.0 software (SPSS Inc., USA).

## Results

### Identification of human BMP2 polymorphisms and genotyping of OPLL and non-OPLL patients

**Figure 2:**
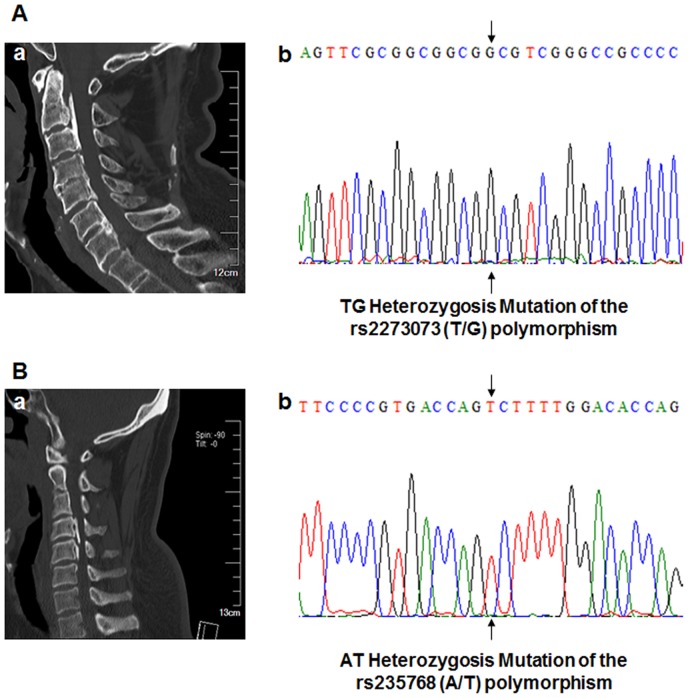
The computerized tomography (CT) and sequencing results of two genotypes of OPLL patients. (A: 55-year-old man; B: 50-year-old woman). (Aa) The CT showed patient had ossification of the posterior longitudinal ligament in the cervical spine (C2-C3, C4-C5, C6-C7, segmental type). (Ab) The direct sequencing result of the PCR products showed the TG heterozygosis mutation of the rs2273073 (T/G) polymorphism of the BMP2 gene. (Ba) The CT showed patient had ossification of the posterior longitudinal ligament in the cervical spine (C4, C4-C5, mixed type). (Bb) The direct sequencing result of the PCR products showed the AT heterozygosis mutation of the rs235768 (A/T) polymorphism of the BMP2 gene.

The two SNPs of rs2273073 (T/G) and rs235768 (A/T) were accurately detected by direct sequencing using BigDye Terminator cycle sequencing on an ABI 3730XL POP7 DNA sequencing analysis 5.2 (Fig. 2). The distribution of genotype and allele type of two SNPs within the BMP2 gene in OPLL patients and controls is shown in Table 3.

**Table 3:** Genotypic and allelic distributions of two SNPs between OPLL cases and controls.

SNPs	rs2273073 (T/G)			rs235768 (A/T)		
Genotype	TT	TG	GG	AA	AT	TT
OPLL (n = 18)	10	8	0	3	10	5
Control (n = 18)	17	1	0	9	7	2
*P* values	*P*<0.05			*P* = 0.64		
Allele	T		G	A		T
OPLL	28		8	16		20
Control	35		1	25		11
*P* values	*P*<0.05			*P*<0.05		

### Morphological changes between the ligament tissues from OPLL and non-OPLL patients

**Figure 3:**
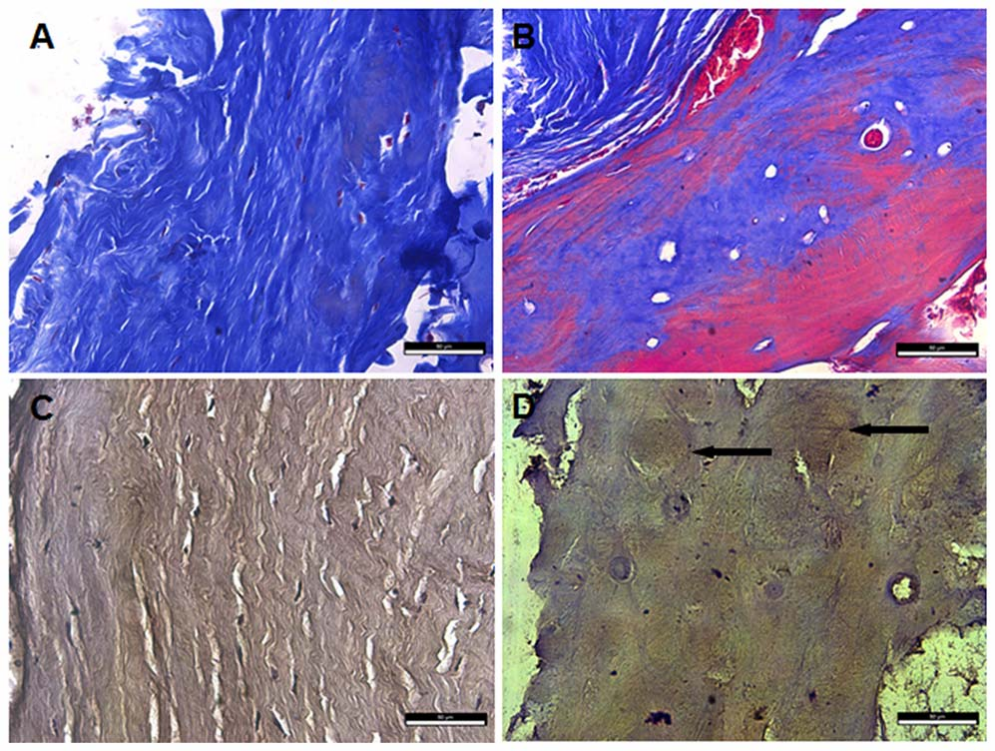
Histological and immunohistochemical examination of ligament tissues from OPLL and non-OPLL patients. (A) Histological examination of ligament tissues from non-OPLL patient, showing normal morphology and texture (Masson staining ×400); (B) Histological examination of ligament tissues from OPLL patient, showing endochondral ossification. Cartilage was stained blue, there were more red dye with the maturing of bone (Masson staining ×400); (C) Ligament tissues from non-OPLL patient with normal structures were un-positive for BMP2 (IHC staining ×400); (D) Ligament tissues from OPLL patient with endochondral ossification structures were positive for BMP2. Representative brown signals are indicated by the arrows (IHC staining ×400). Scale bar = 50 um in all.

We performed histological analysis of ligament tissues in the control group and the OPLL group by Masson Trichrome staining. In the normal ligament tissues, the fibers were arranged in order, with normal morphology and texture, and consistent with the longitudinal direction of the ligaments. Among these fibers, a few fibroblasts appeared and were parallel to the orientation of fibers (Fig. 3A). However, in the OPLL group, fibroproliferative tissue, endochondral ossification tissue and mature bone were found. In the ossification front, some hypertrophic metaplastic chondrocytes were noted in the ossifying plaque immediately contiguous to the ligament fibers, together with a considerable degree of neovascularization (Fig. 3B).

### Distribution of BMP2 in the tissue of the ligaments from OPLL and non-OPLL patients

Immunohistochemical localization of BMP2 was examined using surgical specimens of ligament tissues from an OPLL patient. BMP2 were highly expressed in metaplastic hypertrophic chondrocytes in the ossification front and were found in early lesions of fibroproliferation near the ossifying plaque (Fig. 3D). In the control group, no immunostaining was observed after using antibodies in the ligament tissues from a non-OPLL patient (Fig. 3C).

### Expressions of BMP2 in the surgical specimens of ligament tissues

**Figure 4:**
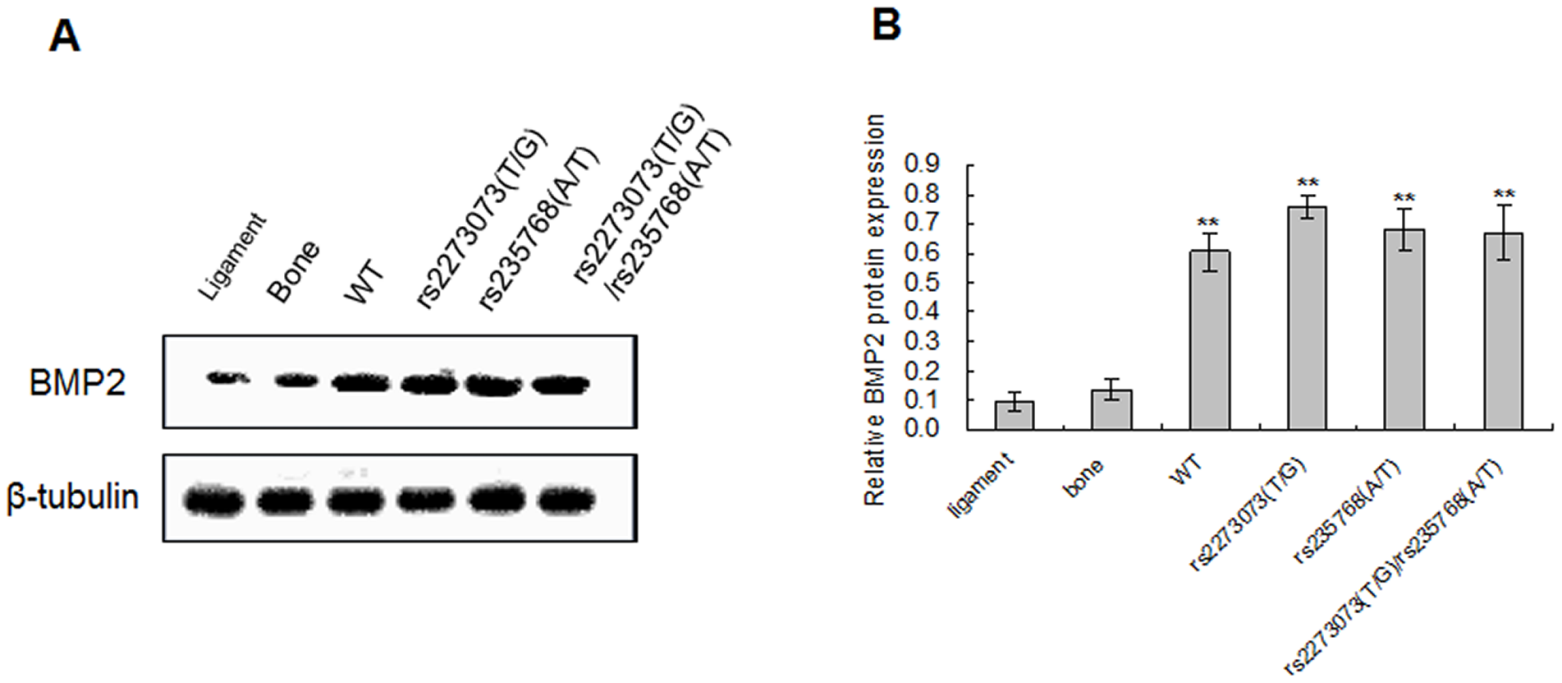
Western blotting analysis of BMP2 protein levels in different groups. (WT: wide-type) (A) BMP2 protein can be detected in all the groups. The ligaments tissues from OPLL patients with different genotypes were showed higher expression. (B) Quantification of BMP2 protein expression in the OPLL patients with different genotypes were higher than the ligament tissues from non-OPLL patients and bone chips from trauma patients, but no statistical difference was seen between each group of themselves. ** *P*<0.01 versus control.

On the basis of genotyping, the ligament tissues of OPLL patients were divided into different groups. As control, the ligament tissues derived from non-OPLL patients and bone chips collected from trauma patients were selected. The expression of BMP2 in each group was detected by Western blotting. It revealed that the ligaments tissues from OPLL patients with different genotypes were significantly higher (*P*<0.01) when compared with the ligament tissues from non-OPLL patients and bone chips from trauma patients, but no statistical difference was seen between each group of OPLL patients with different genotypes (*P*>0.05) (Fig. 4).

### Effects of cyclic stretch on morphology changes of the transfected C3H10T1/2 cells

**Figure 5:**
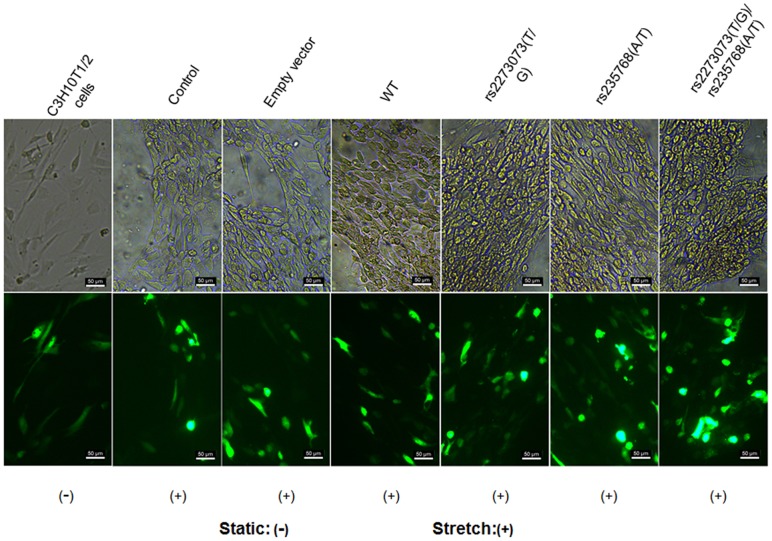
Morphology changes of the transfected cells after mechanical stress. (WT: wide-type) The stretched cells (b-g and i-n) aggregated in different degree along the direction of force and manifested to the osteoblastic differentiation when compared with the static cells (a and h). (a-g) were observed through white light vision and (h-n) were the same view through evaluation for GFP expression with fluorescence inversion microscope. Scale bar = 50 mm in all.

To investigate the effect of cyclic stretch on osteogenic differentiation in the cells with different BMP2 gene variants, we used an extension ratio of 10% and a frequency of 0.5 Hz for mechanical stress to approximate the physical motion of the cervical spine and to avoid damage in the cells. In the presence of mechanical stress, the cells in all the groups aggregated along the direction of force. No significant difference in cellular senescence was observed between static groups and stretched groups. Among the stretched groups, the C3H10T1/2 cells transfected by wild-type or mutant-type expression vectors aggregated more intensely and gradually manifested to the osteoblastic differentiation (Fig. 5).

### Effects of cyclic stretch on expressions of BMP2 in transfected C3H10T1/2 cells

**Figure 6:**
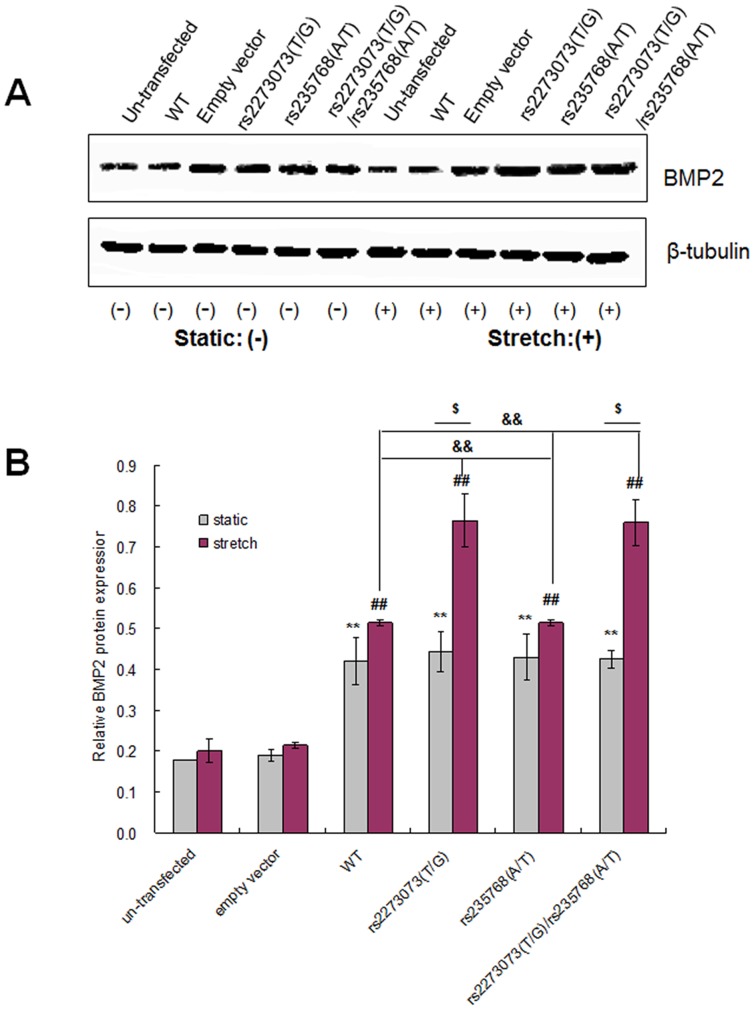
Western blotting analysis of BMP2 protein levels induced by uniaxial cyclic stretch in different groups. (WT: wide-type) (A) BMP2 protein can be detected in all the groups and the expression levels were up regulated after mechanical stress was applied. (B) Quantification of BMP2 protein expression in all the groups. The expression of BMP2 protein in the C3H10T1/2 cells transfected by pcDNA3.1-BMP2 (rs2273073 (T/G)) and pcDNA3.1-BMP2 (rs2273073 (T/G), rs235768 (A/T)) were significantly higher than the other stretched groups (*P*<0.01) and the corresponding static groups (*P*<0.05). **P<0.01 versus un-transfected in the static condition; ##*P*<0.01 versus un-transfected in the stretched condition; &&*P*<0.01 versus the other stretched groups; $ *P*<0.05 versus the corresponding static groups.

We next wondered whether the level of BMP2 expression was affected by uniaxial cyclic stretch. The expression of BMP2 in the stably transfected C3H10T1/2 cells was assayed using Western blotting and it revealed that the expression of BMP2 protein transfected by wild-type or different mutant-type expression vectors was significantly higher than the un-transfected group (*P*<0.01), but there was no statistical difference between them (*P*>0.05) in the static condition. In the presence of mechanical stress, the expression of BMP2 transfected by wild-type or mutant-type expression vectors was higher than the un-transfected group (*P*<0.01). Meantime, the expression of BMP2 transfected by pcDNA3.1-BMP2 (rs2273073 (T/G)) and pcDNA3.1-BMP2 (rs2273073 (T/G), rs235768 (A/T)) were significantly higher than the other stretched groups (*P*<0.01) and the corresponding static groups (*P*<0.05). β-tubulin as an internal standard for cell protein did not change significantly by uniaxial cyclic stretch in either groups (Fig. 6).

## Discussion

In the present study, we firstly revaluated the previously result by investigating the phenomenon in a cohort of 36 patients. The statistical analysis was consistent with previous study which demonstrated that the “TG” genotype of the SNP rs2273073 and the “AT” genotype of the SNP rs235768 were associated with the susceptibility of OPLL again (Table 3). In order to study the probably mechanism of this relationship, ligament tissues from OPLL patients showed enchondral ossification by histological examination and the expression of BMP2 were significantly higher by immunohistochemistry and Western blotting analysis compared with the non-OPLL patients. These results demonstrated that BMP2 was critical for endochondral bone development in OPLL. Next, we established the difference of sensibility to mechanical stretch between different BMP2 gene variants. The C3H10T1/2 cells model with different BMP2 gene variants were constructed and subjected to mechanical stretch. The results suggested the expression of BMP2 transfected by wild-type or mutant-type expression vectors was significantly higher than the un-transfected group, but there was no statistical difference between them in the static condition (Fig. 6). These results were consistent with that of ligament tissues from OPLL patients with different genotypes (Fig. 4). Meantime, in the presence of mechanical stress, the C3H10T1/2 cells transfected by wild-type or mutant-type expression vectors aggregated more intensely and gradually manifested to the osteoblastic differentiation (Fig. 5) and the expression of BMP2 protein transfected by pcDNA3.1-BMP2 (rs2273073 (T/G)) and pcDNA3.1-BMP2 (rs2273073 (T/G), rs235768 (A/T)) were significantly higher than the corresponding static groups (Fig. 6). The existing research results suggested the BMP2 gene variant of rs2273073 (T/G) increased sensibility to the mechanical stress which could affect the cellular morphology, promote the osteogenic differentiation.

Ossification of the posterior longitudinal ligament (OPLL) is a significantly critical pathology that can eventually cause serious myelopathy. OPLL was first reported by Key [34] and later by Oppenheimer [35]. However, this disease attracted our attentions as a cause of myelopathy only after the report by Tsukimoto in 1960 [36]. The involvement of multiple etiologic factors in the development of OPLL has been suggested, including genetic factors, metabolic abnormalities, dietary habits, and some local factors [23], [37]. There are numerous clinical studies about the progression of OPLL under mechanical stress [3], [31]. OPLL cells have been transformed into cells that are highly sensitive to mechanical stress, which may induce the progression of OPLL [38]. OPLL cells can detect and transduce mechanical stretch into biochemical signals that can modulate locomotion [39]. Meanwhile, research suggested stretch-dependent Ca^2+^ influx through SA channels was essential in the stretch-dependent cell orientation and elongation which contributed to the progression of ossification [40]. In present study, we observed the similar effects of cyclic stretch on morphology changes of the C3H10T1/2 cells and our experimental results are supported by those mentioned above.

BMPs was first discovered and described by Marshall Urist [41]. BMP2 gene, located on chromosome 20p12, encompasses 2 exons with a coding region of 1191 nucleotides, produces a protein molecule of 396 amino acids that belongs to the TGF-β superfamily and induces cartilage and bone formation. In the present study, immunohistochemical localization of BMP2 was examined using ligament tissues from the OPLL and non-OPLL patients, the results were consistent with the previous studies that BMP2 were present at the ossifying matrix and chondrocytes of ossifying ligaments, and also localized at mesenchymal cells adjacent to these areas [42]. At the same time, we demonstrated the BMP2 was expressed significantly higher in the OPLL patients through Western blotting which was consistent with the immunohistochemisty observations.

Functional impacts of the BMP2 variants on the OPLL phenotype are currently obscure due to the lack of biochemical evidences. Genetic variations must fulfill two criteria to be considered candidate modifiers of OPLL: the corresponding gene encodes a protein involved in ectopic ossification of OPLL, and the nucleotide change affects gene expression and/or function. In hundreds of cases, a single DNA sequence polymorphism affecting a protein coding sequence has been linked to a clear simple Mendelian phenotype and, for a much smaller but increasing number of cases, to more complex phenotypes. These findings highlight the importance of better understanding the mechanisms leading to inter-individual differences in gene expression in humans. In current study, the Western blotting results suggested in the presence of mechanical stress, the expression of BMP2 protein transfected by pcDNA3.1-BMP2 (rs2273073 (T/G)) and pcDNA3.1-BMP2 (rs2273073 (T/G), rs235768 (A/T)) were significantly higher than the corresponding static groups. BMP2 as secreted proteins guide proliferation and differentiation of mesenchymal cells into bone cells by increasing alkaline phophatase (ALP) and stimulating DNA and procollagen Type I carboxyl-terminal peptide synthesis [43]–[47]. In our previous study, the rs2273073 (T/G) polymorphism of the BMP2 gene is positively associated with the level of Smad4 protein expression and the activity of ALP as Smad mediated signaling pathway plays an important role during the pathological process of OPLL [21]. Meanwhile, RUNX2 is a key regulator of osteocalcin (OCN) and ALP gene promoters and cooperates with BMP-specific Smads, which are important signal transducers regulated in osteoblast differentiation [48]. Furthermore, in vitro and in vivo studies have demonstrated that low-intensity pulsed ultrasound (LIPUS) promoted the bone remodeling by stimulating the HGF (Hepatocyte growth factor)/RUNX2/BMP2 signaling pathway and receptor activator of NFkB ligand (RANKL) expression, and LIPUS increased BMP2 expression via RUNX2 regulation [49]. The existing research results suggests the BMP2 gene variant of rs2273073 (T/G) increases individual sensibility to the mechanical stress which can affect the cellular morphology, promote the osteogenic differentiation.

Our study has numerous limitations. First, although the C3H10T1/2 cell has been utilized as a surrogate stem cell model for the human embryonic fibroblast to study molecular mechanisms of stem cell commitment and differentiation to osteoblast, adipocyte and chondrocyte, there exhibits a similar but not completely identical phenotype which may lead to deviation phenomenon. Second, we didn't detect the expression of other osteogenic genes, such as ALP, RUNX2, COLA1, OPL and OCN, so that it is difficult to understand other susceptible genes and their pathogenetic relevance. Furthermore, although variantions of two SNPs in the BMP2 gene were associated with an elevated incidence of OPLL, the detailed mechanism by which it occur remains obscure. Further investigation is needed to pinpoint the specific function of the BMP2 in the development of OPLL.

In conclusion, these data may provide some insights into the role of mechanical stress in the ectopic bone formation in OPLL. We observed that mechanical stress could increase the expression of BMP2 protein in the C3H10T1/2 cells transfected by BMP2 (rs2273073 (T/G)) and BMP2 (rs2273073 (T/G), rs235768 (A/T)) when compared with the other stretched groups and the corresponding static groups. Based on these observations, we propose that the BMP2 gene variant of rs2273073 (T/G) can not only increase individual susceptibility to OPLL, but also increase the sensibility to mechanical stress which may play an important role during the pathological process of OPLL. These findings may not only contribute to the prevention of progression or recurrence, but also conduce to the postoperative recovery of OPLL in patients with BMP2 gene variant of rs2273073 (T/G). Nevertheless, to clarify the detailed mechanism of BMP2 gene variant in the progression of OPLL, more direct evidences and researches are needed.
